# Benchmarking Standard and Micromechanical Models for Creep and Shrinkage of Concrete Relevant for Nuclear Power Plants

**DOI:** 10.3390/ma16206751

**Published:** 2023-10-18

**Authors:** Vít Šmilauer, Lenka Dohnalová, Milan Jirásek, Julien Sanahuja, Suresh Seetharam, Saeid Babaei

**Affiliations:** 1 Department of Mechanics, Faculty of Civil Engineering, Czech Technical University in Prague, Thákurova 7, 166 29 Prague, Czech Republic; lenka.dohnalova@fsv.cvut.cz (L.D.); milan.jirasek@cvut.cz (M.J.); 2 EDF Lab–Département MMC, Site des Renardières–Avenue des Renardières–Ecuelles, 77818 Moret sur Loing, France; julien.sanahuja@edf.fr; 3 SCK CEN, Engineered and Geosystems Analysis Unit, Waste and Disposal Expert Group, Boeretang 200, 2400 Mol, Belgium; suresh.seetharam@sckcen.be (S.S.); saeid.babaei@vito.be (S.B.)

**Keywords:** autogenous shrinkage, drying shrinkage, total shrinkage, basic creep, total creep, benchmark, models, micromechanics, database

## Abstract

The creep and shrinkage of concrete play important roles for many nuclear power plant (NPP) and engineering structures. This paper benchmarks the standard and micromechanical models using a revamped and appended Northwestern University database of laboratory creep and shrinkage data with 4663 data sets. The benchmarking takes into account relevant concretes and conditions for NPPs using 781 plausible data sets and 1417 problematic data sets, which cover together 47% of the experimental data sets in the database. The B3, B4, and EC2 models were compared using the coefficient of variation of error (CoV) adjusted for the same significance for short-term and long-term measurements. The B4 model shows the lowest variations for autogenous shrinkage and basic and total creep, while the EC2 model performs slightly better for drying and total shrinkage. In addition, confidence levels at 5, 10, 90, and 95% are quantified in every decade. Two micromechanical models, *Vi(CA)${}_{2}$T* and SCK CEN, use continuum micromechanics for the mean field homogenization and thermodynamics of the water–pore structure interaction. Validations are carried out for the 28-day Young’s modulus of concrete, basic creep compliance, and drying shrinkage of paste and concrete. The *Vi(CA)${}_{2}$T* model is the second best model for the 28-day Young’s modulus and the basic creep problematic data sets. The SCK CEN micromechanical model provides good prediction for drying shrinkage.

## 1. Introduction

Concrete structures play an important role for the safe and reliable operation of nuclear power plants (NPPs). Based on the specific NPP type, safety-related concrete structures may include the containment building (foundation, slabs, walls, single-/double-walled dome), containment internal structures (reactor support structures, biological shield, pool structures), reactor buildings, fuel storage pools, cooling towers, etc. [1]. The majority of concrete elements operate in mild environmental exposure conditions at low radiation and at temperatures below 65 °C [2].

Older NPPs were usually designed for a service life of 30–40 years. It became clear that an extension becomes possible with effective aging management, which can ensure safety margins over a prolonged lifetime [1]. The degradation analysis of concrete containment buildings revealed that concrete cracking is a concern in 59% of all reported degradation events ([1], Table 37). The cause of concrete cracking has been attributed by 10% to concrete creep and by 54% to concrete shrinkage ([1], Figure 194). Another classification showed that 51% of all aging problems occurred during the construction stage and 37% during the design phase ([1], Figure 21). This leads to the estimate that correct design for creep and shrinkage helps to mitigate at least 0.37×(0.59+0.10)=26% of degradation events.

Post-tensioned concrete containment buildings guarantee structural integrity in the case of a loss-of-coolant accident (LOCA), sustaining the design pressure of the containment typically in the range of 0.39–0.65 MPa [3]. Concrete creep and shrinkage together with steel relaxation lead to prestress loss, which consequently may induce crack development, leakage, and failure [4]. A recent analysis of 150 containment structures aged 3–40 years showed that the majority had a prestress loss that was smaller than predicted [1]. However, many containment structures exhibited a higher loss, which is attributed to a higher ambient temperature, creep, and shrinkage than anticipated in the design phase [1].

In 2014, Électricité de France (EDF) launched an experimental program called VeRCoRs (VErification Réaliste du COnfinement des RéacteurS) with the one objective of extending the service life of concrete containment buildings. An instrumented, one-third-scaled containment mock-up was built; the EDF has announced three benchmarks for best modeling the practices for leak tightness, early-age modeling, creep modeling, and mechanical behavior during pressure tests [5,6]. The importance of creep and shrinkage have led to the testing of hundreds of samples of VeRCoRs concrete, and the most fundamental tests are benchmarked in this paper.

Radiation-shielding concrete belongs to specific groups in nuclear and radioactive waste storage facilities and takes advantage of heavyweight aggregates for neutron, X-ray, and γ-ray attenuation [7,8]. Experimental data on the creep and shrinkage of such concretes are scarce; however, the radiation-induced volumetric expansion of an aggregate was identified as a first-order mechanism, which was counteracted by the creep of cement paste and damage in a micromechanical model [9,10]. Furthermore, the benchmark only selects concrete with no irradiation exposure, representing the vast majority of NPPs under operation conditions.

According to the classification proposed in [11], the sensitivity of NPP structures to creep and shrinkage was recognized to be at the highest levels, between four and five, for which it is recommended to also perform an analysis of the 95% statistical confidence limits. Common conditions occurring in NPP concrete structures encompass isothermal temperatures of 10–65 °C with drying under an ambient relative humidity of 30–100%. Post-tensioned containment buildings often experience biaxial compressive stress.

The first objective of this paper lies in testing distinct models and modeling approaches for benchmarking the creep and shrinkage behavior of concrete, particularly:

- Standard creep and shrinkage models, namely B3 [12], B4 [13], and the new EC2 [14];
- Micromechanical-based models, particularly
  – *Vi(CA)${}_{2}$T* model for the Young’s modulus and basic creep prediction [15];
  – SCK CEN’s multi-mechanism model for drying shrinkage [16].

The second objective of this paper focuses on micromechanical creep modeling that has never been exploited against large data sets and where practical application raises several concerns. The multi-scale modeling of concrete has brought significant progress in the last decade for civil and material engineering [17] with an extension for creep studies.

Concrete mixes can be highly variable from site to site due to local raw materials. Furthermore, concrete is by design a multi-scale porous material, where multi-physics processes occur. Thus, multi-scale models represent an appealing option to derive physics-informed behaviors and properties. Indeed, such approaches, which are based on homogenization or micromechanics, are able to bridge the scale hosting the physical processes to the scale of interest for the engineer responsible for the structural analysis. In other words, these techniques allow us to step down to the scale where intricate behaviors and couplings that are macroscopically observed can be physically investigated and possibly uncoupled. Another interesting feature of multi-scale modeling is the possibility to break down concrete into individual phases whose intrinsic properties can be expected to remain the same.

The micromechanical modeling of creep starts from the main nano- or micro-mechanisms responsible for this behavior as observed on the macroscopic scale. As consensus has not yet been reached, many hypotheses can be found in the literature [18], such as transfer between capillary and adsorbed water [19], water transfers to newly created microcracks [20], and C-S-H sheets encountering viscous sliding [21]. As this debate regarding the relevant creep mechanisms seems far from being closed, the development of the *Vi(CA)${}_{2}$T* operational toolbox required a pragmatic assumption of the sheet sliding mechanism within C-S-H. Thus, transforming the elastic multi-scale model into a basic creep one only involves the modification of the elementary behavior of C-S-H bricks. A Maxwell model for the strain mechanisms that activate sheet sliding has been considered as a first step [22,23] so that the corresponding characteristic time is the only extra parameter with respect to the elastic model.

Micromechanical modeling of drying shrinkage requires a conceptual understanding of the thermodynamics of pore water in nano-, meso-, and macropores in the hardened cement paste microstructure. Recent approaches hypothesize the existence of multiple mechanisms at different pore scales [24,25,26,27,28,29]. Note that all of these approaches are relevant for reversible drying shrinkage strains, which can be extended using empirically deduced irreversible shrinkage [16]. Three potential benefits of such micromechanical models are (i) an improved understanding of the role of different pore classes to the drying shrinkage process; (ii) prediction of the drying shrinkage strain of cement paste and concrete purely from cement composition and a known conceptual model of the microstructure; and (iii) optimization of mix design for a given application, as well as the prediction of drying shrinkage parameters for historical concrete structures such as NPPs, for which drying shrinkage parameters are unavailable but the mix design is known.

The benchmarking proposed in this paper originates from the revamped Northwestern University creep and shrinkage database, coined in 1976 by Z. P. Bažant. This largest currently available database has been exploited in numerous studies [13,30,31,32], and its significant parts were used for calibrating the B3, B4, and EC2 models. In 2021, the database was revamped into the MySQL 8.0 format with the addition of new experimental data sets [33]. The benchmark also shows the 5, 10, 90, and 95% quantiles of the absolute error for each time decade in order to qualitatively estimate the scatter. Other databases are available with partially overlapping data, such as the Taiwanese database [34] or Japanese database [35].

## 2. Materials

### 2.1. NPP Concretes and Conditions

According to the authors’ experience, the majority of NPP concretes are characterized by mix design, compressive strength, and exposure conditions within the following limits:

- Water/cement ratio 0.30 ≤w/c≤ 0.65;
- Cement mass 250 ≤c≤ 450 kg/m${}^{3}$;
- Cylinder mean compressive strength at 28 days 20 MPa ≤$f_{cm,cyl,28}$≤ 80 MPa;
- Relative humidity 0.30 ≤ RH ≤ 1.0;
- Constant temperature 0 °C < T ≤ 65 °C.

Ordinary Portland cement represents the vast majority of cement in existing NPPs. Occasionally, several blended cements and mineral admixtures have been used in recent decades, e.g., high-strength concrete with a 28-day strength of 64.5 MPa was cast in the massive NPP Civaux-2 in France with a cement content of 266 kg/m${}^{3}$ plus silica fume and calcareous filler [36]. Other examples include concrete with 10–30% fly ash replacement in Ohi’s containment, Japan, with a 28-day strength of 45.88 MPa, or the 40% fly ash substitution used in the main buildings of Sizewell-B in Soffolk, UK, with a characteristic strength of 45 MPa [3].

### 2.2. VeRCoRs Concrete

The 1/3-scaled containment uses VeRCoRs concrete (see Table 1) with cement Gaurain CEM I 52.5 N and mineral mass fractions of 0.52, 0.21, 0.10, and 0.15 for C${}_{3}$S, C${}_{2}$S, C${}_{3}$A, and C${}_{4}$AF, respectively. The concrete has w/c=0.525, and its database entry is M_id =3907. Section 4.8 discusses the benchmark results for the drying shrinkage and basic and total creep.

## 3. Creep and Shrinkage Models

### 3.1. Strain Decomposition in the Standard B3, B4, and EC2 Models

Three well-established models have been selected for benchmarking. The B3 model was published in 1995 as a RILEM standard recommendation [12,37]. Its successor, the B4 model, appeared in 2015 [13]. The last compared model, EC2, was released as FprEN 1992-1-1:2022 [14].

The three above-mentioned models use the shrinkage strain’s decomposition into autogenous and drying shrinkage components as follows:

(1) $$\begin{array}{c} \varepsilon_{sh,total}\left(t,t_{0}\right)=\varepsilon_{sh,aut}\left(t\right)+\varepsilon_{sh,drying}\left(t,t_{0}\right), \end{array}$$

where the B3 model neglects the autogenous shrinkage and EC2 refers to the autogenous shrinkage as the basic shrinkage. The current age of concrete, *t*, represents the time at which the shrinkage strain is evaluated, and $t_{0}$ is the age at the onset of drying.

Deformations due to creep are described by the creep compliance function in the general form below:

(2) $$\begin{array}{c} J\left(t,t^{\prime }\right)=J_{bc}\left(t,t^{\prime }\right)+J_{dc}\left(t,t^{\prime },t_{0}\right), \end{array}$$

where $J_{bc}\left(t,t^{\prime }\right)$ stands for the basic creep compliance and $J_{dc}\left(t,t^{\prime },t_{0}\right)$ stands for the drying creep compliance. The compliance function describes the evolution of the stress-related strain (including the initial elastic strain) per unit of applied stress at loading age $t^{\prime }$. Meanwhile, the EC2 model expresses the compliances using a creep coefficient.

### 3.2. Micromechanical Vi(CA)${}_{2}$T Model

*Vi(CA)${}_{2}$T*, a Virtual Cement and Concrete Ageing Analysis Toolbox, is an internal software tool that was initially dedicated to concrete developed under EDF’s R&D. Its main purpose is to investigate cementitious materials’ properties and behavior through upscaling approaches. These techniques are able to bridge the spatial scale where the physical processes occur to the scale of interest for the engineer. Another interesting feature of upscaling approaches is the possibility to break down concretes into material phases whose elementary properties are expected to remain the same independent of the specific concrete at stake. The *Vi(CA)${}_{2}$T* toolbox is thus a helpful tool for estimating physical properties (such as the evolution of the hydration degree, hydration heat, or capillary porosity), mechanical properties (such as the evolution of the Young’s modulus and basic creep compliance), and transport properties (such as the influence of water content on dielectric permittivity). The toolbox takes into account the concrete microstructure in a material point, and it is not designed to consider structure-scale effects such as the moisture profile that arises during drying. It embeds two types of models:

- Cement hydration models to estimate the evolutions of the amounts of each cement paste phase based on the initial mix design information and the aging conditions, combining the chemistry and kinetics of the hydration reactions;
- Homogenization models to estimate the effective properties and behaviors based on the volume fractions and elementary behaviours of phases and morphological models using upscaling techniques.

The tool, currently ported to Python 3, focuses on generic upscaling models which can be used way beyond cement-based materials. It is thus designed as a generic package that includes several modules implementing physical models. These can be freely combined by the user depending on the intended application. The core modules of homogenization and hydration are briefly described, providing references for more details on the implemented models.

The homogenization module for linear properties is the core of the tool; it estimates the effective properties of a composite material from its phases, along with the phases volume fractions and a description of the composite material morphology. The available linear properties are the elastic stiffness and transport properties such as the dielectric permittivity. The viscoelastic behavior can be investigated via the correspondence principle, allowing us to reuse the linear elasticity homogenization tools through Laplace–Carson transforms. The microstructure modeling module translates a simplified morphological representation to a homogenization scheme, which can be used by the homogenization module to derive estimates of properties.

The hydration module provides estimates of the time evolution of the cement paste constituents depending on aging conditions. Hydration is modeled through simplified kinetics approaches for clinker components (C${}_{3}$S, C${}_{2}$S, C${}_{3}$A, C${}_{4}$AF + gypsum [38,39]), silica fume [40], and blast furnace slag [41]. Stoichiometry is enforced through commonly used, simplified hydration reactions of clinker components, silica fume [42], and blast furnace slag [43]. Together with the heat conservation equation, this simplified modeling yields an initial value problem involving a system of first-order ordinary differential equations. These equations are numerically integrated using backward differentiation formulas (provided by the variable-coefficient Ordinary Differential Equation solver [44]), which is suited for stiff problems.

The homogenization models, which estimate the effective properties and behavior, use the volume fraction and elementary behavior of each phase as inputs, as well as a morphological model describing the geometrical arrangement of these phases. Concrete is described as aggregates embedded into mortar, which is in turn considered as sand grains embedded into cement paste. The morphology of cement paste, which is much less straightforward to the model, builds upon [45] to detail the diverse anhydrous and hydrated phases [46], taking into account the separation of hydrates into low/high-density ones. Two linear upscaling modules are implemented for elasticity and transport. The latter can, for example, be used to homogenize dielectric permittivity. The basic creep behavior is modeled through linear viscoelasticity, assuming that the microstructure does not evolve after the stress or strain loading step. The correspondence principle [47,48] classically takes advantage of the Laplace–Carson transform, which changes any non-aging linear viscoelastic behavior into an elastic one. This allows us to reuse the elasticity upscaling module. Then, the Laplace–Carson transform is numerically inverted using the Gaver–Stehfest algorithm [49,50,51] to obtain the effective compliance or relaxation functions in the time domain.

Validation was performed through benchmarks participation in the framework of the European COST action TU 1404 “Towards the next generation of standards for service life of cement-based materials and structures” [52]. The first two benchmarks were with respect to the hydration and early age stiffness of cement pastes at w/c=0.3 and w/c=0.4, which were predicted from the mix design; see details in [53,54]. The third benchmark was about the non-aging basic creep [55], exercising both the length-scale bridging (predicting the early-age short-term creep of mortar and concretes from early-age short-term creep results on cement pastes) and time-scale bridging (predicting the creep of mature paste from early-age short-term creep results on pastes). The basic creep compliance of C-S-H bricks was identified [55] for early-age short-term creep results on pastes, as provided by the benchmark and as measured using the procedure [56].

Because *Vi(CA)${}_{2}$T* is a material point tool that does not consider drying effects, the mechanical properties that can be estimated by its current version are the Young’s modulus at 28 days and the basic creep compliance. Both are estimated from the same morphological model by considering either elastic or visco-elastic C-S-H particles. The *Vi(CA)${}_{2}$T* model developed for the creep benchmark [55] of COST action TU1404 is reused here as the first approximation. Thus, the same hydration and morphological models and the same intrinsic properties and behavior of phases are considered. In particular, this means that generic aggregate properties and a generic cement (as details on cement are only available for 1.15% of mixes in the database) are used to model all selected mixes of the database. Regarding aggregates, an improvement would be to consider properties that depend on the available mineralogical composition. More details on modeling approaches, validation strategies, and applications are available [15,46,57,58,59].

### 3.3. Micromechanical SCK CEN Model for Drying Shrinkage

The drying shrinkage modeling framework adopted in this study is based on a multi-mechanism approach originally proposed by Powers [60], who presented a thermodynamic analysis of the volumetric shrinkage strain of hardened cement paste attributable to surface free energy, disjoining pressure, and capillary pressure that operate at different relative humidity ranges, which are directly associated with the underlying pore size heterogeneity. Their thermodynamic analysis relates the change in the Gibbs free energy to the water content in different pore classes via Kelvin’s law. Thus, water content in different pore classes forms a key input, which in this study is extracted from a multi-scale water sorption isotherm framework that integrates (i) particle packing, (ii) cement hydration kinetics, and (iii) pore network models [61]. The details of the drying shrinkage framework are described in [16]. Some salient features are recalled here.

The total drying shrinkage strain

(3) $$\begin{array}{ccc} \varepsilon_{v} & = & \varepsilon_{v,r}+\varepsilon_{v,irr}, \end{array}$$

includes both reversible and irreversible components. The reversible shrinkage strain

(4) $$\begin{array}{ccc} \varepsilon_{v,r} & = & \varepsilon_{vc}+\varepsilon_{vs}+\varepsilon_{vd}, \end{array}$$

is the sum of strains due to capillary forces ($\varepsilon_{vc}$), surface free energy ($\varepsilon_{vs}$), and disjoining pressure ($\varepsilon_{vd}$). Strain $\varepsilon_{vc}$ can be derived from Bishop’s single effective stress constitutive equation [62]; $\varepsilon_{vs}$ is similar to Pinson’s approach [27], which in essence is the Bangham equation [63]; and $\varepsilon_{vd}$ follows Powers’s thermodynamic relationship, which essentially integrates water content in pores that are smaller than 2.75 nm, where disjoining pressure is expected to manifest.

There are no thermodynamic relationships as above for determining the irreversible shrinkage, which may be attributed to the densification of LD C-S-H or microcracks. Therefore, Babaei et al. [16] proposed a phenomenological relationship

(5) $$\begin{array}{c} \varepsilon_{v,irr}=\left(\varepsilon_{vs}V_{C\text{-}S\text{-}H}V_{LD\;C\text{-}S\text{-}H}\right)/\eta_{t}, \end{array}$$

to estimate the irreversible strain based on drying shrinkage experiments on six OPC pastes. Here, $V_{C\text{-}S\text{-}H}$ is the volume fraction of C-S-H, $V_{LD\;C\text{-}S\text{-}H}$ is the volume fraction of LD C-S-H, and $\eta_{t}$ is the total porosity.

The above formulation is valid for cement paste alone. However, it can be upscaled to the concrete level using various homogenization techniques (e.g., analytical or numerical), provided that the aggregate size distribution and volume fraction of the concrete are known. In this study, the formulation derived from the analytical homogenization technique by Xi and Jennings (Equation (12) in [64]) is invoked, leading to

(6) $$\begin{array}{c} \frac{\varepsilon_{\mathrm{eff}}^{\mathrm{sh}}}{\varepsilon_{2}^{\mathrm{sh}}}=1-\frac{c_{12}\left(K_{1}/K_{2}\right)}{1+\left(K_{1}/K_{2}-1\right)\frac{3+4c_{12}G_{2}/K_{2}}{3+4G_{2}/K_{2}}}, \end{array}$$

where $\varepsilon_{\mathrm{eff}}^{\mathrm{sh}}$ is the effective shrinkage strain of concrete, $\varepsilon_{2}^{\mathrm{sh}}$ is the shrinkage strain of cement paste, $c_{12}$ is the volume fraction of aggregates, K and G are the bulk and shear modulus, respectively, and subscripts 1 and 2 refer to the aggregates and cement paste, respectively.

Note that the performance of the aforementioned water sorption isotherm [61] and drying shrinkage [16] models has already been tested against existing experimental results for at least nine cementitious materials.

### 3.4. Coefficient of Variation of Error

The deviations of creep and shrinkage model predictions from experimental data are quantified by the coefficient of variation of error [11,30,32]. Short-term and long-term data should have the same significance, which could be achieved by assigning weights that are inversely proportional to the data density. In this paper, time data are subdivided into *n* boxes of equal importance, with each box representing a time decade, i.e., $10^{-3}-10^{-2}$, $10^{-2}-10^{-1}$, …, in days [65] (p. 522). The standard error of regression for large enough data sets is evaluated as

(7) $$\begin{array}{c} s=\sqrt{\frac{1}{n}\sum_{i=1}^{n}\frac{1}{m_{i}}\sum_{j=1}^{m_{i}}{\left(y_{ij}-Y_{ij}\right)}^{2}}, \end{array}$$

where $m_{i}$, i=1,2,…n is the number of data points in a box number *i*, $y_{ij}$ is the measured creep or shrinkage value, and $Y_{ij}$ is the corresponding model prediction. Only time values above $10^{-3}$ days are considered, and the upper limit results from the experimental data.

The weighted mean of all measured values reads

(8) $$\begin{array}{c} \bar{y}=\frac{1}{n}\sum_{i=1}^{n}\frac{1}{m_{i}}\sum_{j=1}^{m_{i}}y_{ij}, \end{array}$$

and the coefficient of variation (CoV) over all data yields

(9) $$\begin{array}{c} \omega =\frac{s}{\bar{y}}. \end{array}$$

## 4. Results and Discussion for Creep and Shrinkage Models

### 4.1. Revamping of the NU Database and Data Credibility

“The Northwestern University Database of Laboratory Creep and Shrinkage Data”, abbreviated as the NU database, presents one of the largest databases in the world of this kind. Currently, the database contains 1468 creep tests with 30,468 data points and 3569 shrinkage tests with 67,590 data points. The data come from 272 published papers/reports and cover 1737 concrete/mortar/paste mixes. A similar cloud-based database exists in Taiwan [34], inheriting several data sets and covering 1400+ sets for creep and 2000+ sets for shrinkage of vibrated concrete. The Japan Society of Civil Engineers (JSCE) published another smaller database [35].

**Figure 1:**
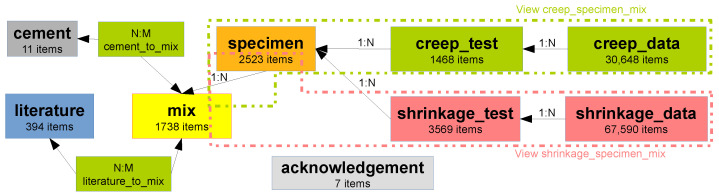
Structure and items in the MySQL version of the NU database.

In 2021, the NU database was revamped into 11 MySQL 8.0 tables with several improvements and fixes (see Figure 1). The relational database uses unique primary keys within each table, which can be referenced to and from another table. The one-to-many relationship (1:N) defines, for example, creep data that belong to a particular creep test carried out on a particular specimen cast from a particular mix. For easier querying, this chain is also present as a view “creep_specimen_mix”. The many-to-many relationship (N:M) joins, for example, the mix and literature data, as one mix can have descriptions in several papers and vice versa.

The database revamp handled several issues covering duplicate items, the introduction of enumerated lists, consistency checks, standard mix design specifications, automatic calculation of various ratios (a/c, w/c), and different cement classifications according to the ASTM, EN, etc. [33]. This revamping process was also an opportunity to better align the database with the FAIR (Findable, Accessible, Interoperable, Repurposable) principles for data management [66], improving the accessibility and searchability of experimental results. Data credibility indicators have been added. MySQL, xlsx versions, and the manual can be downloaded from the Zenodo repository https://doi.org/10.5281/zenodo.8150176 (accessed on 7 October 2023).

Based on the Northwestern University categorization and further detailed analysis, each test is now labeled as PLausible/PRoblematic/ERroneous. For example, problematic data for total creep have no information as to whether drying and autogenous shrinkage have been subtracted. Erroneous data in the total creep miss, instantaneous deformation for example, have a decreasing compliance function during loading or have a non-smooth evolution. The labels are stored in specific columns *T_creepType_CTU* and *ST_shrinkageType_CTU* in order to filter the data. In several cases, it is also unclear whether the amount of water in the mix represents the added water or, correctly, the effective water content.

Data separation into plausible/problematic/erroneous groups is aimed at increasing data credibility. However, aleatoric (stochastic) uncertainty is present in all measured data and epistemic (systematic) uncertainty originates from, e.g., different lab protocols and conditions, device accuracy, or neglection of autogenous shrinkage. Scatter also reflects the historical conditions and might be useful for analyses of older structures.

**Figure 2:**
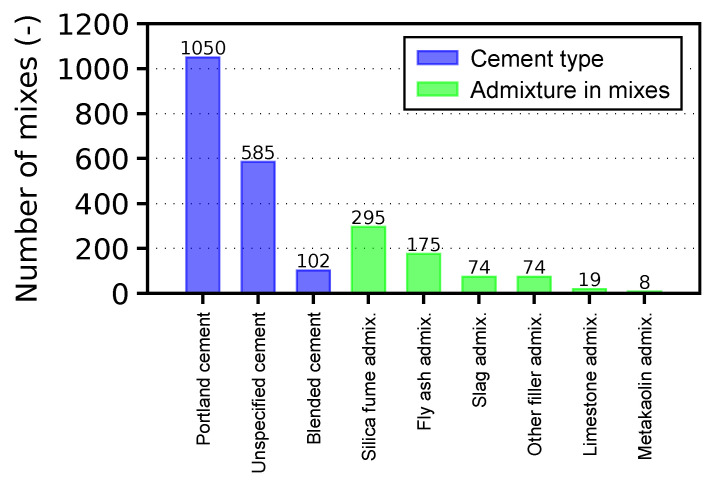
Database statistics in terms of used cement and mineral admixtures.

The database covers 1737 mixes, which can be approximately divided into 86 cement pastes (with cement content $m_{c}>1000$ kg/m${}^{3}$), 259 mortars ($500<m_{c}\leq 1000$ kg/m${}^{3}$), and 1373 concretes ($m_{c}\leq 500$ kg/m${}^{3}$) as distinguished by the cement mass for simplicity. There are 1050 mixes that are declared as Portland cement, 585 unspecified cements that most likely belong to the Portland type, and 102 blended cements (see Figure 2). Several mixes include mineral admixtures as well, mainly containing silica fume and fly ash. Nowadays, blended cement dominates the market (73% of the market in Europe in 2010 [67]) with the need to update creep and shrinkage models. The database is prepared for appending such data.

**Figure 3:**
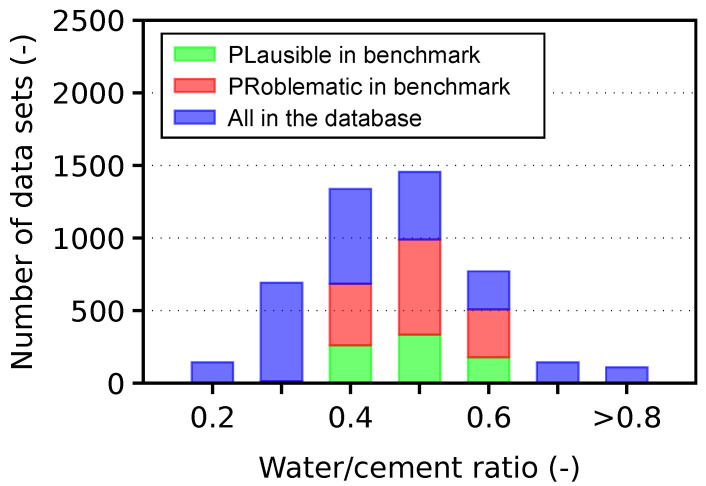
Database statistics in terms of water/cement ratio.

The database contains 4671 PLausible and PRoblematic data sets for any creep and shrinkage test. The scope of the material and conditions, defined in Section 2.1, led to 781 PLausible and 1417 PRoblematic data sets used in the benchmark, covering 47% of all data sets in the database. Figure 3 shows the data sets distribution with respect to the water/cement ratio.

### 4.2. Young’s Modulus at 28 Days

The database contains 173 concrete mixes for which measurements of the Young’s modulus at the age of 28 days are available, taking into account the data restraints described in Section 2.1. The Young’s modulus at 28 days is predicted as 1/J(28+0.001d,28d) from the B3, B4, and EC2 models, where a short-time loading of 0.001 days recovers an instantaneous elastic and short-term creep. The experimental modulus remains intentionally hidden to the models so that, instead, they use the compressive strength at 28 days for the modulus estimate. Otherwise, the models show almost perfect predictions. *Vi(CA)${}_{2}$T* uses a constant elastic stiffness for all aggregates and the same hydration model for all cements.

**Figure 4:**
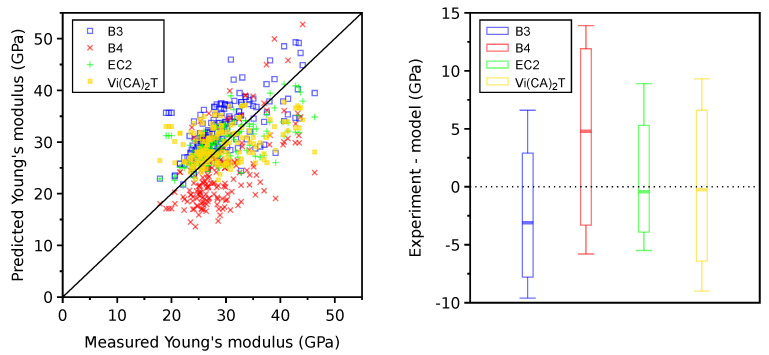
Benchmarking Young’s modulus at 28 days on 173 mixes (**left**) and with 5, 10, 50, 90, 95% quantiles of absolute error (**right**).

The benchmark showed that the EC2 model yields the lowest CoV, followed by the *Vi(CA)${}_{2}$T*, B3, and B4 models; see Figure 4 and Table 2. The candlesticks and whiskerbars in Figure 4 represent the 5%, 10%, 50%, 90%, and 95% quantiles of the absolute error, i.e., $\varepsilon_{experiment}-\varepsilon_{model}$. On average, the B3 model overestimates the modulus by 3.1 GPa and B4 underestimates it by 4.8 GPa, while EC2 and *Vi(CA)${}_{2}$T* exhibit no systematic bias. The CoV from *Vi(CA)${}_{2}$T* is comparable with the other tested models and shows a high potential of the micromechanical model. The experimental scatter originates above all from experimental errors and different procedures, variations in aggregate stiffness, and differences in curing conditions across laboratories.

It is interesting to extend the mix selection to all database data that specify cement mass, water mass, total aggregate mass, and Young’s modulus at 28 days. In this case, the selection yields 597 mixes out of 1738, including pastes, mortars, and concretes. The *Vi(CA)${}_{2}$T* model gives CoV =0.23 and the absolute errors are −16,−12,3.1,4.9,7.4 GPa for the 5,10,50,90,95% quantiles, respectively. Extending the selection led to CoV increasing from 0.18 to 0.23, still showing a high predictive potential of the *Vi(CA)${}_{2}$T* model, covering even pastes and mortars.

### 4.3. Autogenous Shrinkage

Experimental data cover 42 plausible data sets with temperatures ranging between 20 and 53 °C. The majority of data correctly assign zero strain to the final setting time approximately between 2 and 9 h of hydration. Several experiments took zero shrinkage at 1 day, skipping the initial part.

**Figure 5:**
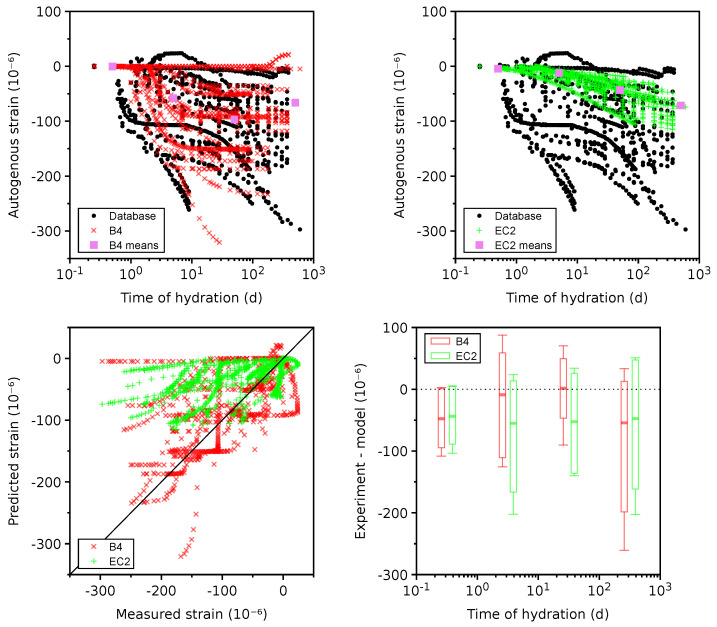
Autogenous shrinkage on 42 plausible data sets.

Figure 5 displays the autogenous shrinkage for two shrinkage models, including the mean and scatter values in each time decade. It is clear that model B4 as well as EC2 underpredict the autogenous shrinkage on average, especially for times above 100 days. A recent analysis of long-term autogenous shrinkage revealed that some mixes have a logarithmic strain evolution with no final value [68]. Even uncontrolled drying shrinkage would not change the trend as it has the final value and is additive. Both the B4 and EC2 models assume a final asymptotic value, which contradicts several long-term experimental data [30].

### 4.4. Drying Shrinkage

**Figure 6:**
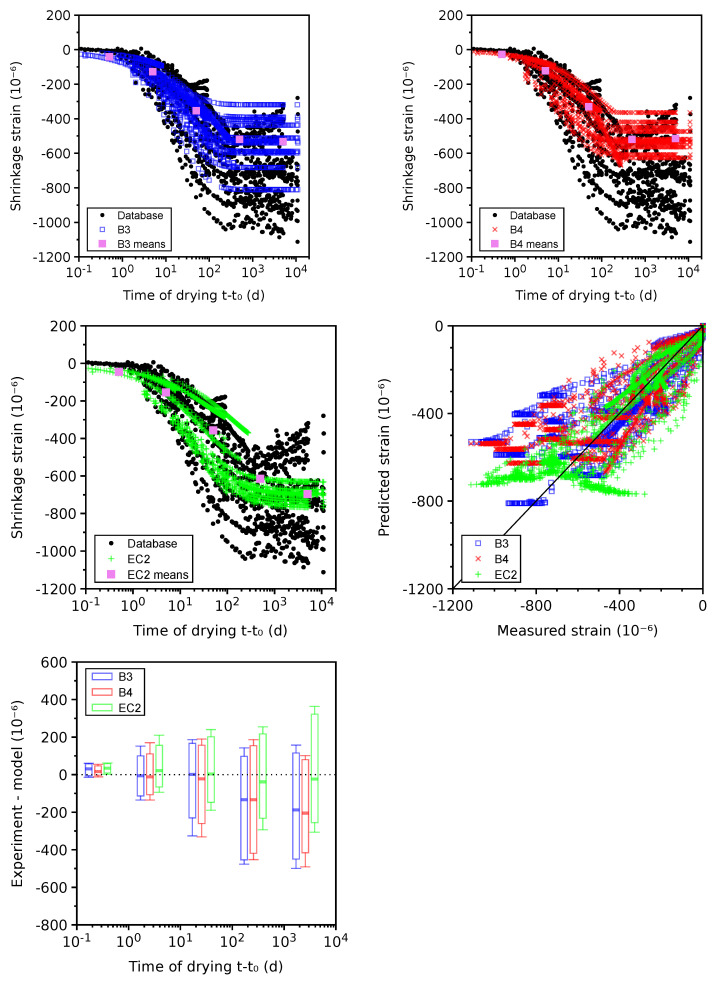
Drying shrinkage on 32 plausible data sets.

Drying shrinkage covers 32 plausible data sets (see Figure 6). All models exhibit a rather symmetric scatter in every decade. The smallest CoV is obtained with the EC2 model (see Table 2), followed by B3 and B4. The B4 model can yield a CoV of 0.46 when the unknown aggregate is replaced by sandstone.

**Figure 7:**
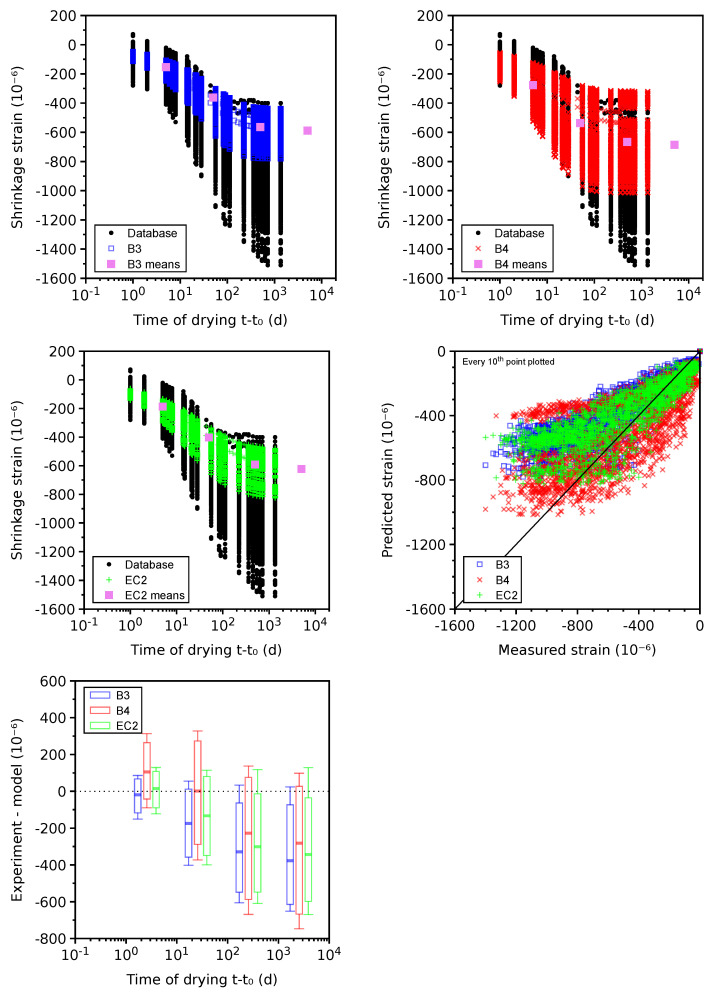
Drying shrinkage on 899 problematic data sets.

Problematic data sets in Figure 7 do not contain experimental points under 1 day of drying or the literature does not mention whether autogenous shrinkage has been subtracted. Taking 899 problematic data sets slightly decreases the CoV of the B3 and B4 models and slightly increases the CoV of the EC2 model.

All models underestimate long-term asymptotic shrinkage on average. When analyzing the decade $t-t_{0}\in \left(10^{3},10^{4}\right)$ days, the means for the B3, B4, and EC2 models are −589, −685, $-623\times 10^{-6}$ with 90% quantile errors of −615, −667, $-598\times 10^{-6}$, respectively. If statistical analyses should be performed at the 90% confidence level, the predicted drying shrinkage values need to be multiplied by a factor of 1.96–2.04 depending on the model. The EC2 model suggests a factor of 1.4 for 90% confidence, which is largely underestimated.

The common origin of long-term underestimation lies in too short drying time, taking typically 1–3 months [69]. Extrapolation is an ill-conditioned problem without knowledge of the shrinkage half-time and asymptotic value. A small companion specimen has been suggested, shifting the drying time by $2\log(D_{1}/D_{2})$, where $D_{1}$ is the original size and $D_{2}$ is the size of the companion specimen [69]. From the micromechanical perspective, a higher elastic modulus of aggregates leads to a higher restraint and lower asymptotic drying shrinkage [30]. The benchmarked results show that 25 out of 110 mixes contain no information about the coarse aggregate type, which increases the scatter of B4 model when taking into account aggregate type.

### 4.5. Total Shrinkage

**Figure 8:**
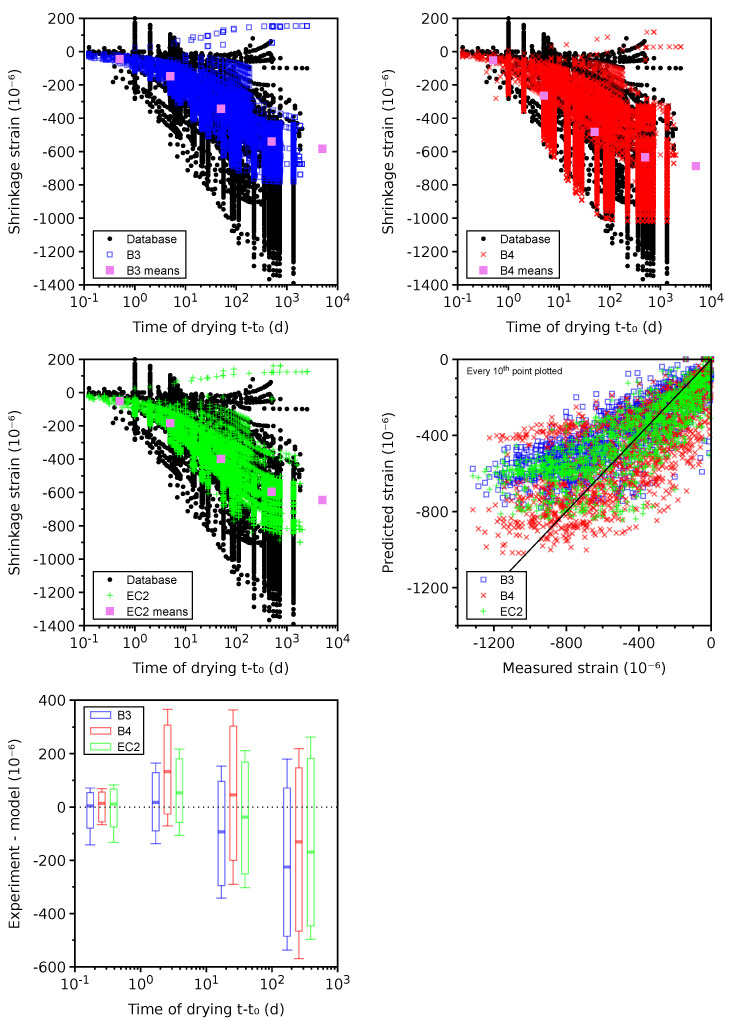
Total shrinkage on 655 plausible data sets.

The total shrinkage is shown for 655 plausible data sets (see Figure 8), skipping 165 problematic data sets leading to similar results. Total shrinkage represents the sum of autogenous and drying shrinkage according to Equation (1). Generally, the models underestimate the long-term values in a similar way to drying shrinkage itself. The majority of data reveals that autogenous shrinkage shows small changes after 200 days; hence, drying shrinkage is responsible for the long-term underestimation. The CoV values between drying and total shrinkage are similar for the same reason.

### 4.6. Basic Creep

**Figure 9:**
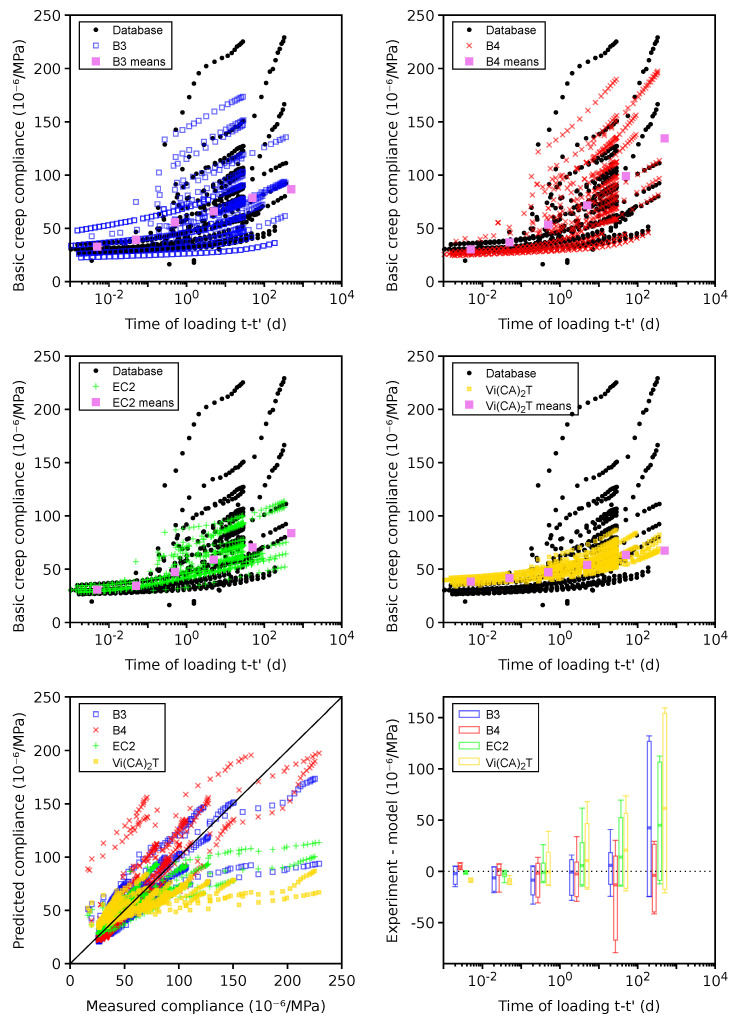
Basic creep on 32 plausible data sets.

Only 32 plausible data sets remained for benchmarking; the other 25 data sets were classified as erroneous due to missing the instantaneous elastic strain in the compliance function. Figure 9 shows the benchmark results. The error demonstrates the systematic underestimation of compliances by the B3, EC2, and *Vi(CA)${}_{2}$T* models for $t-t_{0}>100$ days, leading to the lowest CoV occurring in the B4 model.

The highest CoV in the *Vi(CA)${}_{2}$T* model is likely attributed to the simplicity of the basic creep model. In particular, generic aggregate properties are used for all of the mixes. Also, the same generic cement is considered for all mixes, leading to the same hydration kinetics except for the w/c influence. Note that the only information input from the database to the *Vi(CA)${}_{2}$T* model is the mix design; no model parameter is calibrated from the available data (neither on the selected data sets nor on the other ones present in the revamped database). The idea of this confrontation to a rather large data set is to set a first basis from which future model improvements can be quantified. Foreseen improvements could include using aggregates’ mineral information to select a more relevant stiffness or using cement type information (even if the detailed cement composition is not available) to choose from several “generic” cements to be defined for each type. Furthermore, the Young’s modulus at 28 days could be used as additional information to identify a mix-dependent parameter.

**Figure 10:**
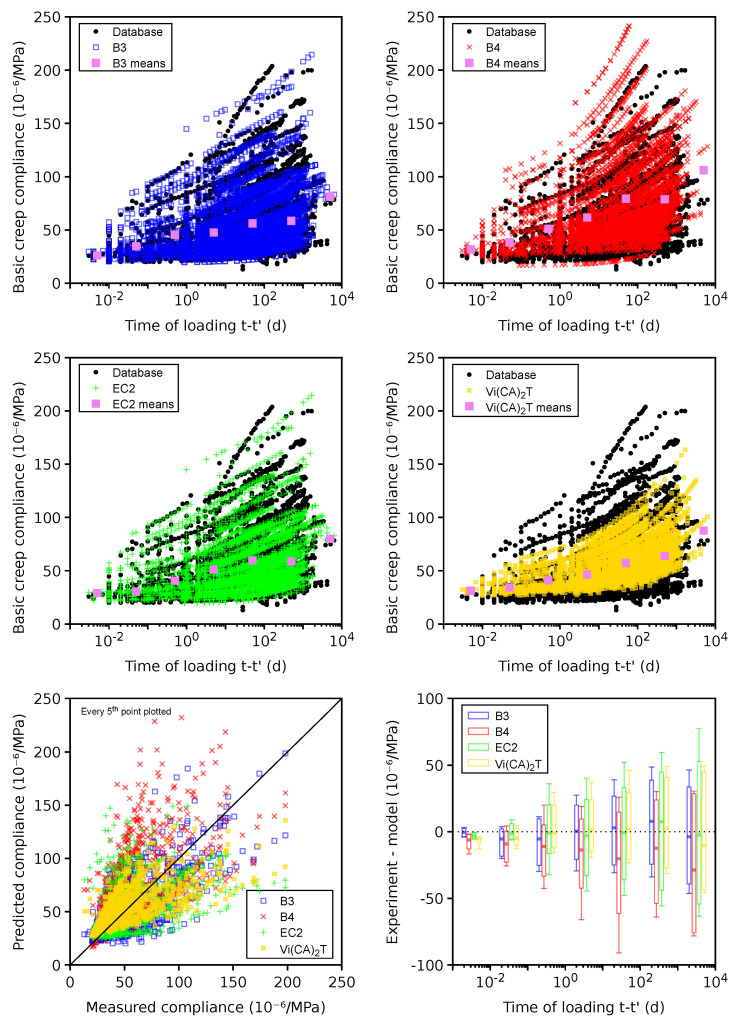
Basic creep on 181 problematic data sets.

Figure 10 gives 181 problematic data sets, which mainly signal the unclear subtraction of autogenous shrinkage from the total strain. Out of them, 143 data sets have w/c<0.50, where autogenous shrinkage is generally non-negligible.

The B4 model tends to overestimate average compliances for $t-t_{0}>100$ days, which likely stems from the missing autogenous shrinkage and from the long-term bridge deflection data used for B4 calibration [32]. A large portion of problematic data were used to calibrate the B3 model, which logically shows the lowest CoV. A comparably low CoV is generated by the *Vi(CA)${}_{2}$T* model, exhibiting an almost zero mean error in all decades. The reason why the *Vi(CA)${}_{2}$T* model seems to behave differently from the case of plausible data sets still has to be investigated.

### 4.7. Total Creep

**Figure 11:**
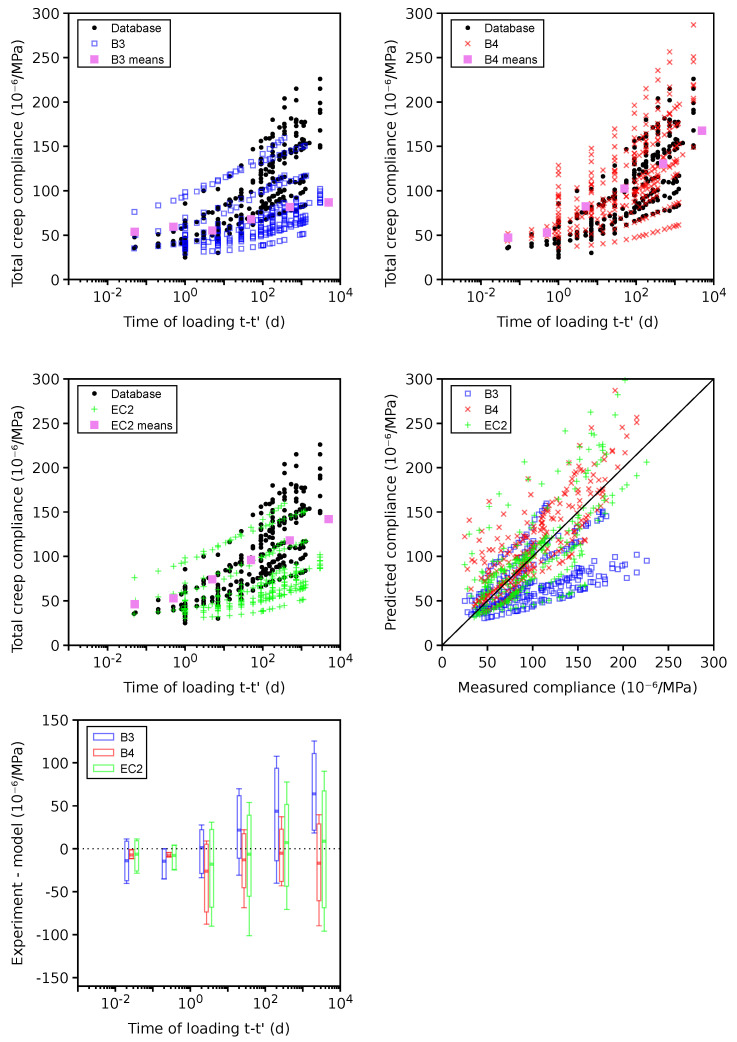
Total creep on 20 plausible data sets.

Plausible data for the total creep contain 20 data sets, 12 of which were previously set as erroneous due to missing the instantaneous elastic compliance. The error in Figure 11 shows the systematic underestimation when the B3 model is used for $t-t^{\prime }>100$ days. Extensive recalibration for the B4 model by also using long-term bridge creep data in addition to the database led to higher long-term total creep and better results [32]. It should be mentioned that external drying leads to drying creep, which significantly contributes to the total creep according to Equation (2). Microcracking, irreversible deformation, and stress-induced shrinkage were identified as possible mechanisms for drying creep; however, the evolution was always calibrated from experiments and its similarity with drying shrinkage [70].

**Figure 12:**
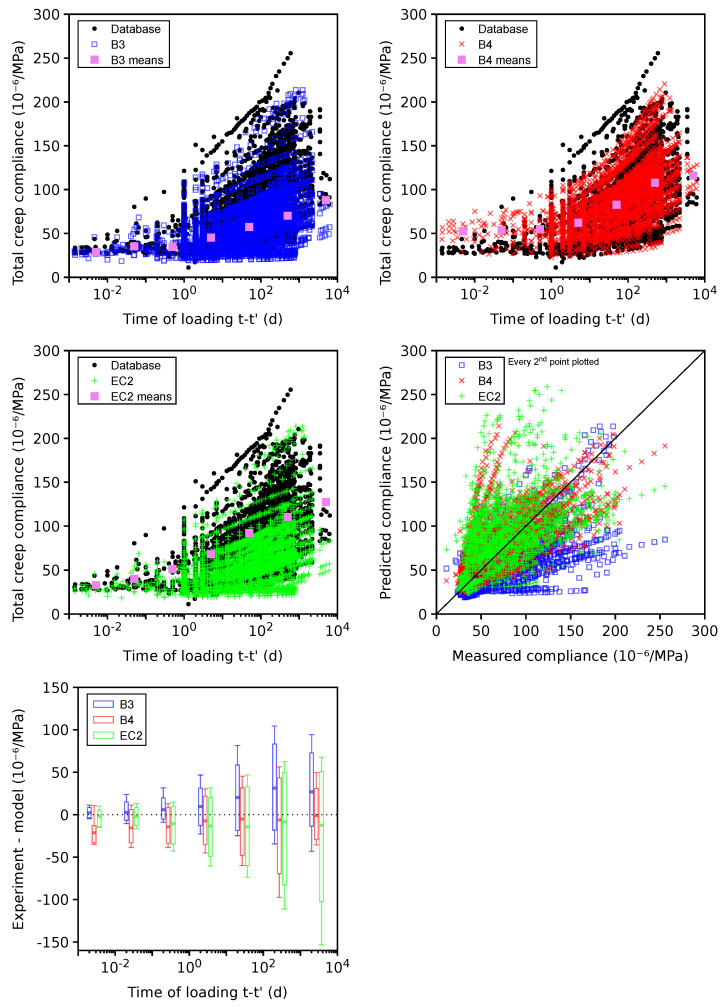
Total creep on 169 problematic data sets.

The 169 problematic data sets show no error on average (see Figure 12). The B4 model yields the lowest CoV for both the plausible and problematic data sets; see Table 2. Apparently, the long-term bridge data also contributed to the best B4 performance [32].

### 4.8. VeRCoRs Concrete

**Figure 13:**
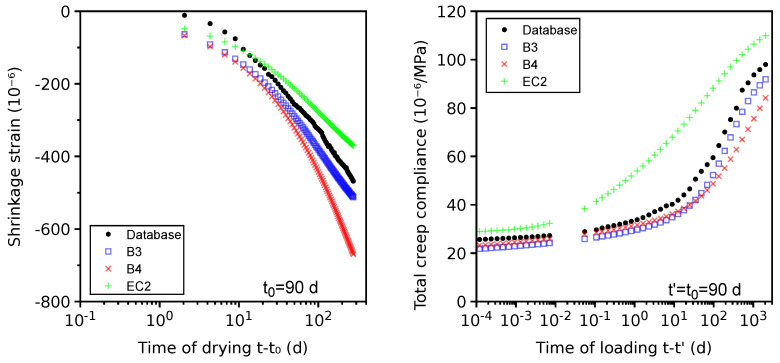
Benchmarking the drying shrinkage (**left**) and total creep (**right**) of VeRCoRs concrete.

The properties of the VeRCoRs concrete were assessed in great detail for the needs of a benchmark of a double-walled containment building [5]. Autogenous shrinkage was measured and found to be negligible as its value was $-19\times 10^{-6}$ at 212 days. Figure 13 shows the drying shrinkage on cylinders (160 mm in diameter and 1000 mm in length) exposed to 48% relative humidity, starting at 90 days (database entry ST_id = 3700). All shrinkage models predict similar behavior. The same specimens were tested for total creep, with drying and loading being started simultaneously at 90 days (database entry CT_id =1470); see Figure 13. All models provide generally good predictions; the EC2 model slightly overestimates compliance between 0.1 and 100 days.

**Figure 14:**
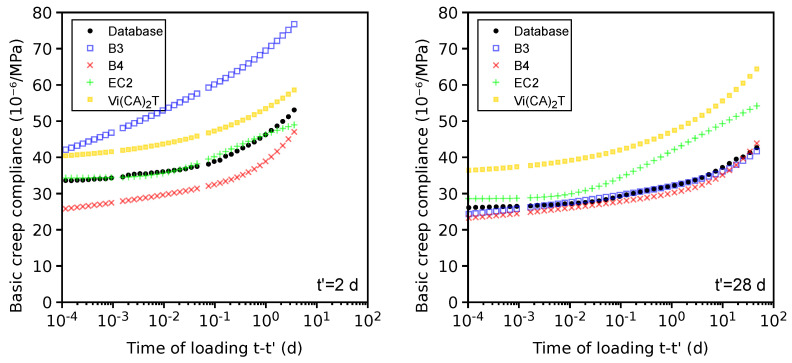
Benchmarking the basic creep of VeRCoRs concrete.

The basic creep for loading at $t^{\prime }=2$ and 28 days is presented in Figure 14 (database entries CT_id =1440 and CT_id =1442). The B3 model shows an exaggerated effect of aging, and the B4 and EC2 models are close to the results. The *Vi(CA)${}_{2}$T* creep model does not take into account the aging effects of hydrates, and the hydration becomes frozen when $t\geq t^{\prime }$. Recent works aim at overcoming this limitation but are not yet integrated into the *Vi(CA)${}_{2}$T* toolbox [71,72].

### 4.9. Differences in Total Shrinkage between Portland and Blended Cements

During the last decades, ordinary Portland cement has decreased its market share substantially; for example, Europe reports only a 27% market share in 2010 [67]. The database has enough data to compare the total shrinkage for concretes constructed from Portland and blended cement.

The similarity was quantified as a 10% relative difference within all of the following parameters: cement mass, water/cement ratio, aggregate/cement ratio, average compressive cylinder strength at 28 days, volume/surface ratio, temperature, relative ambient humidity, and time of the onset of drying.

Mixes of the compared sets contained no additional mineral admixtures beyond those used directly in the cement. Problematic data sets were included as they represent only 19% and according to Table 2 show no significant difference in the coefficient of variation. There are 55 data sets for OPC concretes, which matched 243 data sets for concretes created from blended cement.

**Figure 15:**
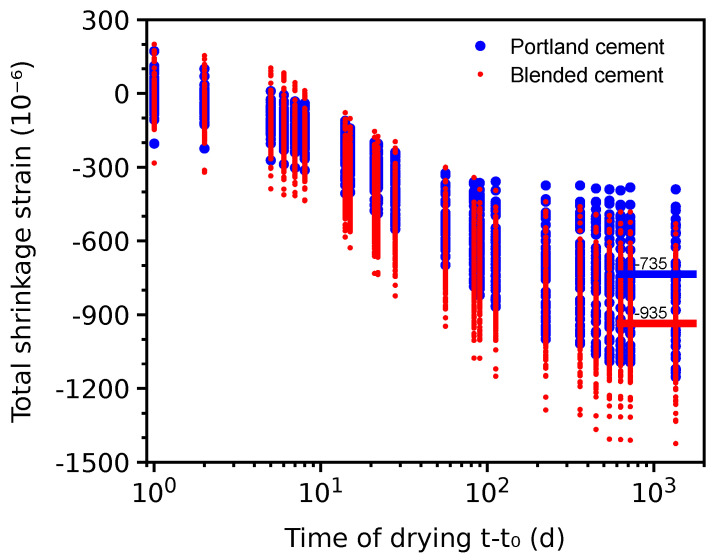
Total shrinkage of Portland and blended cement on mutually comparable data sets.

Figure 15 shows the total shrinkage evolution for both groups. Shrinkage after 600 days came close to asymptotic values and the averages are −735 and $-935\times 10^{-6}$ for both groups, which is a factor of 1.27. Preserving similar strength evolution with less reactive mineral materials is commonly facilitated by a higher Blaine fineness. A larger average shrinkage of blended cement is likely caused by finer particles creating smaller pores and higher capillary pressure [73]. An increase in drying shrinkage with finer cement was experimentally demonstrated [74]. A change in the drying kinetics in blended cement was noticed by T. Sakthivel et al. [75], who adjusted parameters of the B4 model.

### 4.10. SCK CEN Model for Drying Shrinkage

The main objective of this analysis is to test the capability of the model to estimate the drying shrinkage strain of cement paste. For this, recent work by Kinda [76] is found to be the most appropriate experimental campaign as it not only addresses the drying shrinkage of the VeRCoRs cement paste material but also considers sample sizes of 200 µm, 500 µm, and 2 mm, which are reasonably close to the material point of view and are hence easier to compare with the model results, which is targeted towards the representative volume element (RVE) analysis. Moreover, an important conclusion from Kinda’s study is that the drying shrinkage is more or less independent of the specimen shape, size, and drying rate for the range of samples that was considered.

**Figure 16:**
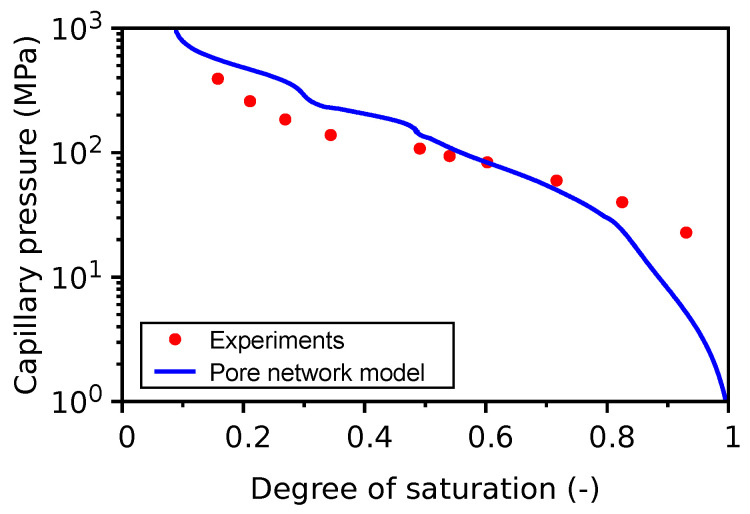
Water desorption isotherm.

VeRCoRs hardened cement paste samples were cured for 3–6 months under endogenous conditions [76]. These were directly subjected to drying after curing; hence, the measured total drying shrinkage included reversible as well as irreversible strains, e.g., due to any cracking or densification of C-S-H. Based on the cement composition in Table 1, cement hydration kinetics are computed using VCCTL [77]. The resultant phase fractions are presented in Table 3. The pore size distribution for gel pores for OPC has been previously estimated (Section 2.1 in [61]). The pore size distribution for pores ≥1 µm has been extracted from VCCTL in this study. The pore size distribution between these two classes of pores is unknown and is hence simply approximated as described in Section 2.3 in [61]. With the estimated total porosity and pore size distribution, the water desorption isotherm has been estimated as shown in Figure 16. The model is able to show good correspondence with the experimental desorption isotherm in the degree of saturation range of 0.5–0.8 but deviates in the lower and higher range of degree of saturation, which is attributed to various assumptions in the particle packing and hydration kinetics conceptual models. In contrast, in a previous validation exercise [61] involving ten different OPC-based cementitious materials, good correspondence was reached for the lower and higher range of degrees of saturation rather than in the intermediate range.

Based on the effective homogenization formulation [64], Table 4 presents the calculated bulk modulus of the cement paste, including a comparison with the experimental values. A deviation of 10% is obtained, which is considered admissible and is hence taken forward in the drying shrinkage calculation. The inputs required for the effective homogenization calculation are available in Table 4.

**Figure 17:**
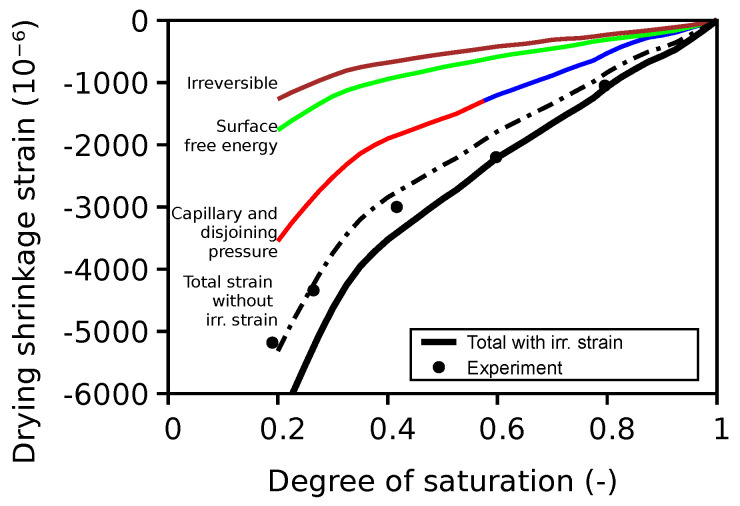
Validation of drying shrinkage strain on cement paste.

A comparison of the estimated versus experimental ultimate drying shrinkage strain is presented in Figure 17. The total strain with irreversible strain gives reasonable predictions in the degree of saturation range from 1 to 0.4, which is typically the exposure range of structural components, but it overestimates the experimental data below 0.4. In contrast, the total strain with no irreversible strain component underestimates at the higher degree of the saturation range but predicts well at lower degrees of saturation. Previous validation experience [16] has shown that without the irreversible strain component, it is not possible to predict the experimental strains well. However, note that the phenomenological relationship Equation (5) for obtaining irreversible shrinkage strain is based on the calibration of limited experimental data, and any uncertainty related to this is reflected in the deviation observed. Equation (5) was derived from experiments on larger-sized samples where cracking may have occurred during drying, especially at very low degrees of saturation. However, in the experiment, the size of the samples was small enough to avoid cracking [76].

To gain deeper insight, the contribution of capillary forces, surface energy, and disjoining pressure are also superimposed in Figure 17. In accordance with the hypothesis discussed in Section 2.3 of [16], the surface free energy is operational at all degrees of saturation, whereas the capillary force is operational from 100% down to 55% and the disjoining pressure is operational under 55%. Figure 17 also shows the contribution from the irreversible shrinkage strain, which is generally less than the three contributions to the reversible strain, and its magnitude is much higher at lower degrees of saturation, which explains the deviation of the estimated results.

**Figure 18:**
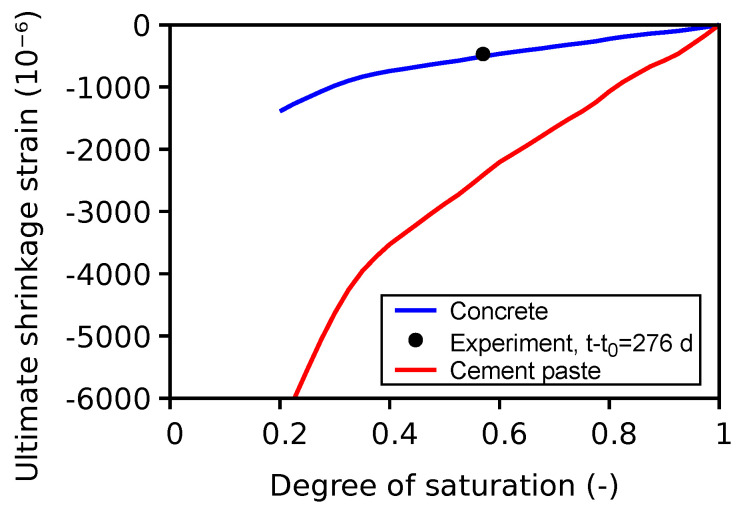
Estimated ultimate shrinkage strain for cement paste and concrete.

The experimental drying shrinkage data for VeRCoRs concrete are presented in Figure 13(left). It is seen that, in the experiment, a steady state has still not been reached, even after 276 days of drying. However, the model can only predict the ultimate drying shrinkage strain. Hence, it is difficult to compare the two sets of data. As discussed in Section 3.3, the volume fraction of aggregates and the shrinkage strain of cement paste for a specific degree of saturation are the key inputs for Equation (4). Note that the ultimate drying shrinkage strain curve with the irreversible strain component is considered for the analytical homogenization. Based on these, Figure 18 shows the estimated drying shrinkage strain for concrete, which is significantly reduced by the presence of aggregates, whose volume fraction is roughly 71% in VeRCoRs concrete. The experimental RH of 50% roughly corresponds to a 57% degree of saturation, which yields the ultimate estimated shrinkage strain of $500\times 10^{-6}$ against experimental $468\times 10^{-6}$ (7% deviation). Note that the upscaling of the shrinkage strain to the concrete scale only takes care of addition of aggregates of a certain volume fraction but not the microcracking, creep effects, or ITZ influence.

Nevertheless, the model shows promising results given that the estimation is performed from mere cement composition data and the synthesis of the existing microstructural understanding of hardened OPC paste.

## 5. Conclusions

Benchmarking the creep and shrinkage behavior of NPP concretes against five models leads to the following conclusions:

1. The EC2 model shows the best prediction for the autogenous, drying, and total shrinkage, both for the plausible and problematic data sets in terms of CoV of error. The EC2 model provides the best prediction of the Young’s modulus at 28 days;
2. The B3 and B4 models exhibit the best performance for the basic and total creep, both for the plausible and problematic data sets;
3. The SCK CEN model for water desorption isotherm yields a reasonably good estimation compared to the experimental results. This is also reflected in the reasonably good estimation of ultimate shrinkage strain, with the model overestimating the shrinkage strain of the cement paste and concrete by ≈20% and ≈7%, respectively.
4. The micromechanical model *Vi(CA)${}_{2}$T* shows the second best prediction for the 28-day Young’s modulus and yields comparable CoV values for the basic creep. It was the first time that a micromechanical model was benchmarked against such large data sets. The model used the constant intrinsic properties of aggregates and the same generic model for cement, which can be improved;
5. Autogenous shrinkage shows in general a high coefficient of variation, which is likely caused by various chemical admixtures. When w/c is approximately below 0.50, the autogenous shrinkage becomes non-negligible and direct measurements are preferred due to low time demand. The question of the final asymptotic value has not been resolved yet [68];
6. Concretes made from blended cement increase the final total shrinkage by a factor of 1.27 on average when matched with comparable Portland cement concretes. Future development needs to take into account the dominance of blended cement and the impact on creep and shrinkage;
7. Although the focus of this paper deals with NPP concretes, the findings are transferable to similar concretes in buildings, bridges, dams, and near surface and deep geological nuclear waste disposals.

## Figures and Tables

**Table 1:** Mix design of the VeRCoRs concrete.

Component	kg/m${}^{3}$
CEM I 52.5 N	314
Effective water	165
Fine aggregate	814
Coarse aggregate	976

**Table 2:** Benchmarking the database for NPP concretes. Coefficient of variation (CoV) for the four models and the PLausible/PRoblematic data sets. Bold value shows the best model for PLausible data sets.

Test Type	PLausible/ PRoblematic	Data Sets	Points	Weighted Mean $\bar{y}$	B3 CoV	B4 CoV	EC2 CoV	$\mathrm{Vi}{\left(\mathrm{CA}\right)}_{2}T$ CoV
Young’s modulus at 28 d	-	173	173	29.29 GPa	0.19	0.26	**0.14**	0.18
Autogenous shrinkage	PL PR	42 3	870 37	−82 $\times \;10^{-6}$ −205 $\times \;10^{-6}$	- -	**0.82** 1.05	0.97 1.37	- -
Drying shrinkage	PL PR	32 899	1344 19,647	−327 $\times \;10^{-6}$ −642 $\times \;10^{-6}$	0.56 0.48	0.56 0.44	**0.43** 0.47	- -
Total shrinkage	PL PR	655 165	15,117 1281	−376 $\times \;10^{-6}$ −340 $\times \;10^{-6}$	0.58 0.55	0.54 0.62	**0.53** 0.46	- -
Basic creep	PL	32	749	64 $\times \;10^{-6}$/MPa	0.46	**0.30**	0.46	0.65
	PR	181	4241	49 $\times \;10^{-6}$/MPa	0.37	0.61	0.54	0.42
Total creep	PL	20	261	85 $\times \;10^{-6}$/MPa	0.50	**0.33**	0.40	-
	PR	169	3262	65 $\times \;10^{-6}$/MPa	0.47	0.43	0.56	-

**Table 3:** Phase fractions of VeRCoRs cement paste from the hydration kinetics calculation.

Parameter	Value
Degree of hydration	0.85
Volume fraction low-density (LD) C-S-H	0.27
Volume fraction high-density (HD) C-S-H	0.28
Total C-S-H	0.55
Capillary porosity	0.21
Total porosity	0.37
Portlandite	0.14
Unhydrated clinker	0.06
Other products	0.04

**Table 4:** Experimental and calculated bulk moduli.

Bulk Modulus	GPa
Experimental ($K_{b}$)	10.5
Calculated ($K_{b}$)	9.6
Calculated solid ($K_{s}$)	17.5

## Data Availability

Creep and shrinkage database (MySQL, xlsx versions, and manual) can be downloaded from the Zenodo repository https://doi.org/10.5281/zenodo.8150176 (accessed on 7 October 2023).
